# Spinal anesthesia for L5-S1 interlaminar endoscopic lumbar discectomy: a retrospective study

**DOI:** 10.1186/s12891-023-06956-z

**Published:** 2023-10-14

**Authors:** Guanyi Liu, Jinsong Zhao, Liyong Yuan, Fangling Shi, Liangguang Zhang

**Affiliations:** 1 Department of Orthopedics, Ningbo No. 6 Hospital, 1059 Zhongshandong Road, Ningbo, Zhejiang 315040 People’s Republic of China; 2 Department of Anesthesiology, Ningbo No. 6 Hospital, 1059 Zhongshandong Road, Ningbo, Zhejiang 315040 People’s Republic of China

**Keywords:** L5-S1 disc herniation, Percutaneous endoscopic interlaminar lumbar discectomy, Spinal anesthesia, Advantages, Complications

## Abstract

**Objective:**

This study aimed to report our experience with spinal anesthesia (SA) in patients undergoing L5-S1 interlaminar endoscopic lumbar discectomy (IELD) and clarify its advantages and disadvantages.

**Methods:**

One hundred twelve patients who underwent IELD for an L5-S1 disc herniation under SA were retrospectively analyzed. SA with 0.5% ropivacaine was administered using a 27-gauge fine needle. Intraoperatively, the volume and level of SA, surgical time, blood loss, and cardiopulmonary complications were documented. Postoperative data was collected included the number of patients who ambulated on the day of surgery, incidence of complications and were then statistically analyzed.

**Results:**

Analgesia was complete throughout the entire operation in all patients and no other adjuvant intraoperative analgesic drugs were needed. Mean visual analog scale scores for intraoperative and early postoperative (24 h) pain were 0 and 2.43 ± 1.66. SA was administered at the L3-4 interspace in 34 patients (30.4%) and the L2-3 interspace in 78 (69.6%). Administration was successful with the first attempt in all patients. Mean operation time was 70.12 ± 6.52 min. Mean intraoperative blood loss volume was 20.71 ± 5.26 ml. Ninety-eight patients ambulated on the same day as surgery. Mean length of hospital stay was 24.36 ± 3.64 h. Dural injury without damaging the nerve root occurred in one patient. One patient experienced recurrent disc herniation. Intraoperative hypotension and respiratory distress occurred in five (4.5%) and three (2.7%) patients, respectively. Three patients (2.7%) received postoperative analgesia therapy and two (1.8%) experienced nausea. Two patients (1.8%) developed urinary retention. Spinal headache, cauda equina syndrome, and neurotoxicity did not occur.

**Conclusion:**

SA can achieve satisfactory pain control for patients undergoing IELD with a low incidence of adverse events. SA may be a useful alternative to local and general anesthesia for IELD surgery. Future randomized controlled trials are warranted to investigate.

## Introduction

Lumbar disc herniation is traditionally treated using open surgery to remove the herniated portion of the nucleus pulposus. Endoscopic transforaminal and interlaminar discectomy techniques have also gained acceptance [1]. Generally, interlaminar endoscopic lumbar discectomy (IELD) is recommended for herniations at the L5-S1 level. The L5-S1 interlaminar space is wide, which is conducive to an interlaminar approach; moreover, the iliac crest typically impairs transforaminal access to the L5-S1 disc space [2]. IELD is also faster than the transforaminal approach and intraoperative radiation exposure is lower [3].

However, the optimal anesthesia technique for IELD is controversial [4–10]. General anesthesia (GA) is routinely used but has been associated with postoperative cognitive dysfunction (POCD) in elderly patients [4]. Other disadvantages include nausea, vomiting, and higher postoperative analgesic requirement, which increase the difficulty of postoperative management. Postoperative hospital stay is also longer after GA [5]. Although local anesthesia (LA) is another option for IELD [6–8], it does not completely eliminate surgery-related pain. Some patients have experienced adverse cardiovascular events caused by unbearable pain during surgery [9, 10].

Lumbar disc surgery may also be performed under spinal anesthesia (SA) [11–13]. In a meta-analysis comparing SA and GA for lumbar spine surgery, the incidence of postoperative nausea and vomiting and volume of intraoperative blood loss were lower and length of stay was shorter in patients who received SA [14]. Attari et al. [15] reported that SA provided better postoperative analgesia and perioperative hemodynamic stability than GA without increasing the incidence of adverse side effects. Although previous studies have shown satisfactory outcomes with SA for conventional open lumbar disc surgery, few have examined the use of SA for IELD [4–15]. This study aimed to report our experience with SA in patients undergoing L5-S1 IELD and clarify its advantages and disadvantages.

## Materials and methods

### Study design and patients

We retrospectively reviewed the medical records and imaging examinations of 122 consecutive patients who underwent L5-S1 IELD under SA at our department between December 2018 and February 2021. The study protocol was approved by the Ethical Review Board of Ningbo No. 6 Hospital. The inclusion criterion was radicular leg pain with magnetic resonance imaging suggesting a single-segment L5-S1 disc herniation, and no response to 4 to 6 weeks of conservative treatment. The exclusion criteria were patients with an L5-S1 interlaminar distance < 7 mm or those who had a history of surgery at L5-S1. SA was not used in patients with contraindications to SA (local infection, coagulopathy) or regional anesthesia in the prone position without a protected airway (morbid obesity, sleep apnea). All patients underwent routine anteroposterior and lateral lumbar spine radiography, computed tomography, and magnetic resonance imaging before surgery. After excluding 10 patients with less than 12 months of follow-up, 112 were included for analysis. Demographic and preoperative clinical features of 112 patients are shown in Table 1.

**Table 1:** Demographic and clinical features of 112 patients

Variable	Value
Age (years)	46.71 ± 10.23
Sex	
Male	70(62.5%)
female	42(37.5%)
Height (cm) weight (kg)	168.30 ± 7.35 67.12 ± 8.62
Hypertension	26 (23.2%)
Diabetes	15 (13.4%)
ASA physical status	
I	34(30.4%)
II	78(69.6%)
III	0
Type of herniation	
Protrusion	70(62.5%)
Extrusion	52(46.42)
Location of herniation	
Central	32(28.57%)
Paramedian	80(71.42%)
Foraminal	0
Surgical time (min)	60.12 ± 6.52
Blood loss (ml)	20.71 ± 5.26

ASA, American Society of Anesthesiologists

### Anesthesia technique

Blood pressure, heart rate, oxygen saturation, and electrocardiography monitoring were initiated once the patient entered the operating room. Inhalational oxygen was delivered at a rate of 3 L/minute. Ringer’s lactate solution was infused via a 20-gauge indwelling needle placed into a peripheral vein at a rate of 8 mL/kg/hour.

**Fig. 1:**
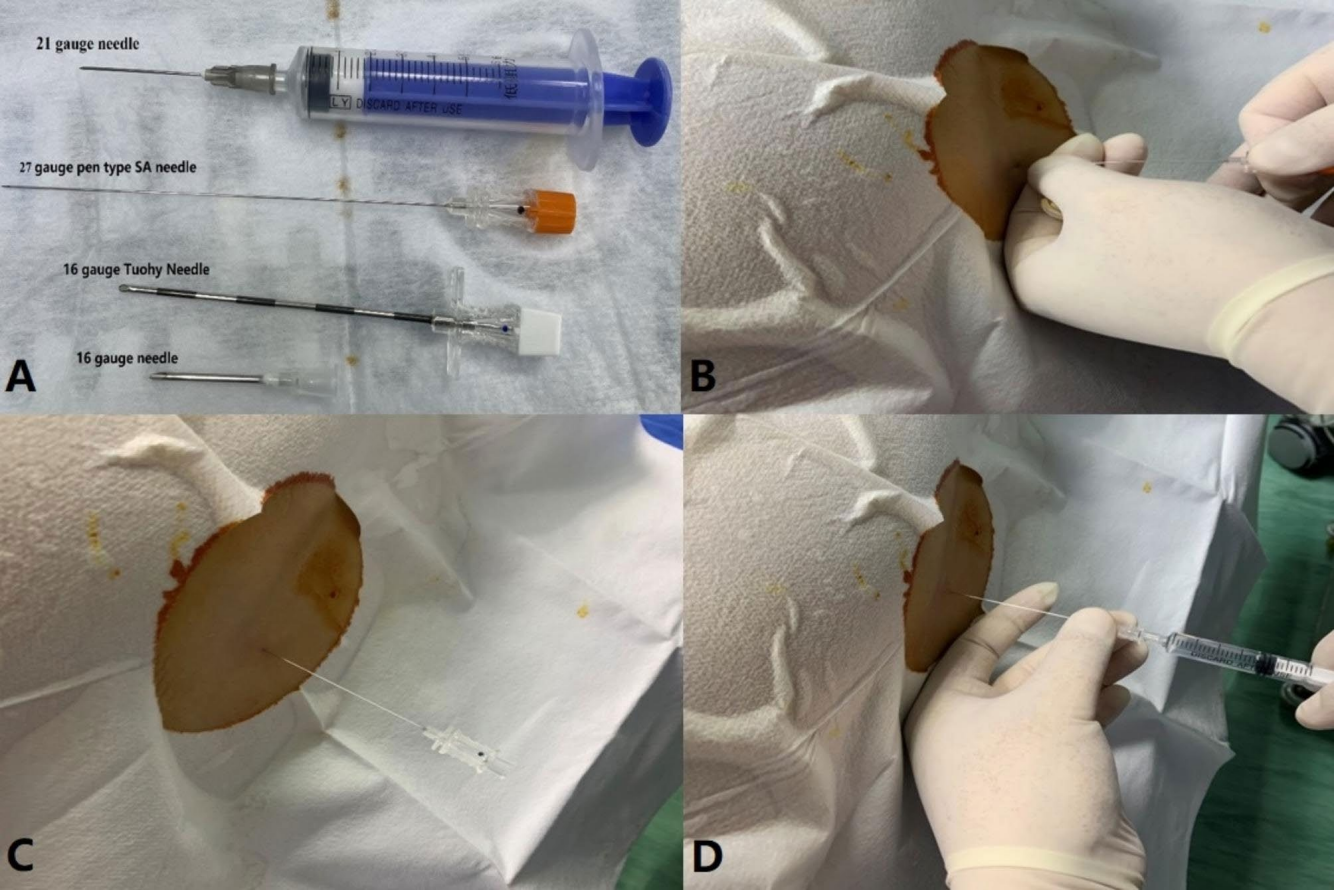
(**A**) Comparison of the 27-gauge spinal anesthesia needle with other needle types. (**B**) Spinal anesthesia puncture. (**C**) Cerebrospinal fluid flows from the core of the spinal needle. (**D**) 0.5% ropivacaine was injected into the subarachnoid space

SA was administered by an experienced anesthetist with the patient in the right lateral decubitus position. All lumbar puncture procedures are performed using a 27-gauge fine Pencil Point needle to reduce the risk of post-spinal headache [16] (Fig. 1A). Lumbar puncture was performed at approximately the L2-3 or L3-4 intervertebral space (Fig. 1B). Once free flow of cerebrospinal fluid was verified through the needle, 2 mL of 0.5% ropivacaine was injected into the subarachnoid space over 5 s (Fig. 1C and D). The sensory level of block was assessed 10 min after SA by pinprick test. Ten minutes later, patients were placed into prone position [17]. Any episodes of intraoperative hypotension (systolic blood pressure < 80% of baseline) were treated with ephedrine 6–12 mg and fluid boluses as needed. Heart rate < 50 beats/minute was treated with atropine 0.5 mg.

### Operative technique

**Fig. 2:**
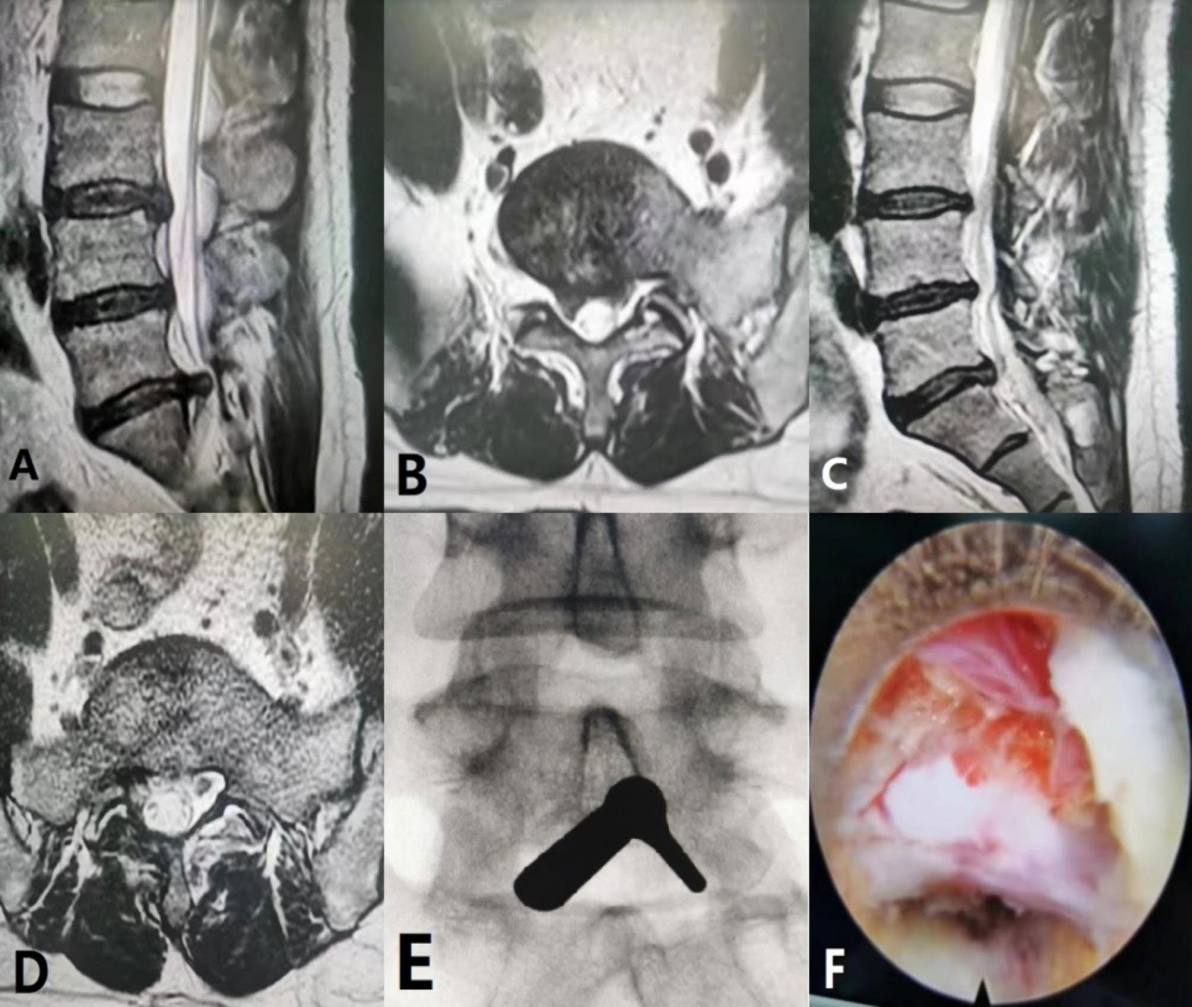
Images of a 40-year-old man who underwent intralaminar endoscopic lumbar discectomy. Preoperative T2-weighted sagittal (**A**) and axial (**B**) magnetic resonance images show an L5-S1 disc herniation. Sagittal (**C**) and axial (**D**) images 2 years after surgery show complete removal of the herniated portion of the disc. Intraoperative images show (**E**) the beveled working sheath at the interlaminar space and (**F**) the S1 nerve root after decompression

IELD was performed with the patient in the prone position under fluoroscopic guidance. The skin incision was made in the midline over the L5-S1 interlaminar window. After dissecting to the fascia, it was incised and a 6 mm dilator was advanced bluntly to the lateral edge of the interlaminar window. Then, a 7 mm operation sheath with a beveled opening was directed toward the ligamentum flavum. The final position was checked on an anteroposterior fluoroscopic image. After introducing an endoscope (Richard Wolf GmbH, Knittlingen, Germany), the lateral border of the ligamentum flavum was exposed and identified under direct visualization and constant irrigation. A lateral 5 mm incision was made in the ligamentum flavum to expose the neural structures. Then, the protruded disc material was excised using the technique described by Ruetten et al. [18]. S1 nerve root decompression was confirmed by observing free movement of nerve root and pulsation of the dural sac. (Figure 2)

### Clinical data and outcomes

The following pre- and intraoperative data were collected: patient age and gender, volume and level of SA, operation time (total time in the operating room including anesthesia time), blood loss volume, and cardiopulmonary complications (e.g. intraoperative hypotension and respiratory depression). Postoperatively, we recorded the incidence of nausea, vomiting, urinary retention, spinal headache, cauda equina syndrome, and other complications. When the muscle strength of both lower limbs returned to level V, the patient was allowed to ambulate and the time was recorded. The visual analog scale (VAS) scores of intraoperative and postoperative analgesia were assessed in all patients.

### Statistical analysis

Statistical analyses were performed using SPSS software version 18.0 (IBM Corp., Armonk, NY USA). Continuous data are presented as means with standard deviation.

Comparisons of preoperative and postoperative values were performed using the paired Student’s t-test. All tests were two-sided. P < 0.05 was considered significant.

## Results

### Patient and clinical data

Mean patient age was 46.7 years. Seventy patients were men and 42 were women. American Society of Anesthesiologists status was I in 34 patients (30.4%) and II in 78 patients (69.6%). Twenty-six patients (23.2%) had hypertension and 15 (13.4%) had diabetes (Table 1). Type of herniation was protrusion in 70 patients (62.5%) and extrusion in 52 patients (46.42).

### Surgical outcomes and complications

Mean operation time was 70.12 ± 6.52 min. Mean intraoperative blood loss volume was 20.71 ± 5.26 ml. Ninety-eight patients (87.5%) ambulated wthin 6.04 ± 1.13 h on the same day after surgery. Mean length of hospital stay was 24.36 ± 3.64 h. Mean postoperative follow-up was 26.27 ± 7.86 months. All patients had effective relief of back pain and leg pain. Dural injury without damaging the nerve root occurred in one patient during ligamentum flavum removal in one patient; the patient’s leg pain improved and no postoperative cerebrospinal fluid leak developed. No neurovascular complications, uncontrolled epidural bleeding, wound infection, or medical complications occurred. One patient (0.08%) experienced recurrent disc herniation 3 months after surgery which was addressed via minimally invasive transforaminal lumbar interbody fusion.

### SA efficacy and Complications

SA was administered at the L3-4 interspace in 34 patients (30.4%) and the L2-3 interspace in 78 (69.6%). Administration was successful with the first attempt in all patients. Analgesia was complete throughout the entire operation in all patients and no other adjuvant intraoperative analgesic drugs were needed. No patient required conversion to GA. Mean visual analog scale scores for intraoperative and early postoperative (24 h) pain were 0 and 2.43 ± 1.66. Transient hypotension occurred after SA administration during the change from supine to prone position in five patients (4.5%). Ephedrine treatment was successful in all. Three patients (2.7%) complained of respiratory distress when they were placed in the prone position, which was relieved after adjusting the chest cushion. Three patients (2.7%) received intramuscular tramadol (100 mg) for incisional pain after surgery and two experienced nausea (1.8%). Urinary retention requiring bladder catheterization occurred in two patients (1.8%); the catheter was successfully removed before discharge in both. Spinal headache, cauda equina syndrome, and neurotoxicity did not occur (Table 2).

**Table 2:** Complications and adverse reactions to spinal anesthesia

Complications Subtype	No. Complications	Incidence Rate	Treatment of complications
Intraoperative hypotension	5	4.5	Ephedrine hydrochloride 6 mg IV
Intraoperative respiratory depression	3	2.7	Adjusting the chest cushion with oxygen 3 L/min inhalation.
Postoperative nausea and vomiting	2	1.8	Metoclopramide 10 mg IV.
Urinary retention	2	1.8	Urethral catheterization
Analgesic requirement	3	2.7	Tramadol 100 mg IM if VAS > 5.
Postdural puncture headache	0	0	Not available
Cauda equina syndrome	0	0	Not available
Neural toxicity	0	0	Not available
POCD	0	0	Not available

IV, injection of vein; IM, injection of muscle; VAS, visual analog scale; POCD, postoperative cognitive dysfunction

## Discussion

Safety and efficacy are fundamental principles to consider when selecting modality of anesthesia. In terms of safety, neurotoxicity, cauda equina syndrome, and spinal headache are serious potential complications of SA. Nneurotoxicity has been reported mostly in association with intrathecal injection of bupivacaine and levobupivacaine [21, 22]. Nevertheless, ropivacaine has been identified smaller neurotoxic potential [23]. Therefore, we selected ropivacaine as an intrathecal drug. Second, we did not add opioid adjuvants such as fentanyl to local anesthetics. It is well known that, local anesthetic adjuvants like opioids were effective in prolonging intraoperative and postoperative analgesia [24]. But IELD is a minimally invasive surgery, and the postoperative back pain is mainly related to the surgical effect, while the surgical incision pain is minor. Too long time of anesthesia is not conducive to the early postoperative evaluation of surgical effects. Moreover, there is also a risk of pruritus and vomiting from the use of local anesthetic adjuvants [25]. Third, we chose 0.5% as the concentration of ropivacaine for intrathecal injection. A lot of studies have shown that ropivacaine has the shortest blocking time compared with bupivacaine and levobupivacaine at the same concentration; 0.5% ropivacaine has significantly shorter anesthesia time than 0.75% ropivacaine [26–31] (Table 3). As mentioned above, the shorter blocking time is consistent with the needs of our study. Therefore, we obtained good anesthetic effects with 0.5% ropivacaine in this study and observed no incidence of neurotoxicity. The mechanisms underlying cauda equina syndrome and spinal headache complications are complex and mainly related to dural puncture by a thick needle [32]. The use of a fine 27-gauge spinal needle can reduce these risks [16, 33, 34]. We observed no incidence of cauda equina syndrome or spinal headache in this study.

**Table 3:** Comparison of anesthesia efficacy and complications of different local anesthetics versus 0.5% ropivacaine

Local anesthetic	Mean time to onset(min)	Duration of sensory block(h)	Duration of motor block(h)	Hypotension (%)	Urinary retention (%)	References
0.5% bupivacaine	—/↓	↑	↑	—/↑		[26],[27],[28]
0.5% levobupivacaine		↑	↑		—	[29], [30]
0.75% ropivacaine	—	↑	↑			[31]

—, no significant difference compared to 0.5% ropivacaine; ↓, significantly lower than 0.5% ropivacaine; ↑, significantly higher than 0.5% ropivacaine

IELD requires greater S1 nerve root retraction and manipulation than the transforaminal discectomy, which can result in severe radicular pain. Therefore, the effectiveness of anesthesia is very important. Ye et al. [20] reported that 66.6% of patients undergoing IELD under LA experienced pain intraoperatively, which was described as unbearable in 20%. Zhang et al. [36] reported that some patients under LA could not tolerate the pain during endoscopic discectomy and required opioid administration during the operation. Airway management of a prone patient who has received opioids can be challenging. In contrast, we found SA to be highly efficacious: intrathecal ropivacaine provided complete intraoperative analgesia in all patients and no additional analgesia was necessary. Ahmed et al. [37] reported that patients undergoing SA for open lumbar disc surgery had a lower incidence of postoperative nausea and vomiting (15.63% vs. 18.36%) and lower analgesic dose requirement in the first 24 h (2.31 ± 0.592 vs. 3.22 ± 0.491) than those undergoing GA. In our study, only three patients (2.7%) required postoperative analgesia and 2 patients (1.8%) had nausea due to postoperative tramadol analgesia.

Operation time and hospital stay were relatively short in our study. Mean operation time was approximately 70 min and mean hospital length of stay was only 24 h. In contrast, reported operation times for IELD using GA range from 70 to 78 min and reported mean lengths of hospital stay range between 3.8 and 4.7 days. For IELD using LA, the corresponding ranges are 50–78 min and 2.9–3.3 days, respectively [10, 20, 38]. Although SA has no significant advantage over GA and LA in terms of operation time, it does appear to be associated with a shorter hospital stay, possibly because of fewer adverse reactions to anesthesia and better postoperative analgesia. De Biase et al. [39] also reported that SA offers advantages over GA in the setting of minimally invasive lumbar surgery in terms of postoperative pain and patient mobilization.

IELD under SA may reduce the risk of POCD. In one study, incidence of POCD after IELD under GA was 42% on day 1 and 16% on day 7 after surgery; in the LA group, POCD was not observed [4]. Although POCD is not fully understood, studies have shown that intraoperative hypotension and delayed ambulation after surgery are associated risk factors [40, 41]. In our patients, the incidence of intraoperative hypotension was very low (4.5%) and a high percentage of patients ambulated on day of surgery (87.5%). These two factors can prevent cognitive dysfunction [42–45]. None of our patients developed POCD (Table 4). In summary, SA can provide complete intraoperative (vs. LA) and early a postoperative analgesia (vs. GA). In addition, there may be significant advantages in early ambulation, discharge time, and prevention of PONV and POCD. However, these potential benefits need to be confirmed by further randomized controlled trials.

**Table 4:** Comparison between other anesthesia methods and SA results in this study

bAnesthesia methods	Intraoperative analgesia VAS ≥ 4 (%)	early postoperative analgesia VAS(≤ 24 h)	Interval time to ambulation (h)	Hospital stay (h)	Intraoperative hypotension (%)	Intraoperative respiratory depression (%)	PONV (%)	Urinary retention (%)	POCD (%)	References
**SA**	0	2.43 ± 1.66	6.04 ± 1.13	24.36 ± 3.64	4.5	2.7	1.8	1.8	0	Our study
**GA**					12.5		18.36			[37]
**GA**				91.92 ± 20.4						[38]
**GA**				112.8 ± 23.04						[20]
**GA**			11.41 ± 2.06	114.24 ± 20.64						[10]
**GA**		4.9 ± 2.3	14.27 ± 5.62	39.3 ± 18.9						[39]
**GA**									16–42	[4]
**LA**	20									[20]
**LA**				71.04 ± 13.92						[38]
**LA**				79.2 ± 11.28						[20]
**LA**			5.50 ± 1.17	71.04 ± 19.68						[10]
**LA**									0	[4]

VAS, visual analog scale; h, hour; PONV, Postoperative nausea and vomiting; POCD, postoperative cognitive dysfunction; SA, spinal **Anesthesia;** d, day; GA, general anesthesia; LA, local anesthesia

Using SA for IELD has potential disadvantages: First, it does not allow determination of intraoperative nerve root injury based on patient feedback. However, recent study found no neurovascular injury during the epidural anesthesia for IELD, the concentration of ropivacaine was 0.29% to preserve maximum tactile sensation for patients with allowed mild pain. This concentration could allow for timely feedback to avoid nerve injury when the nerve root is contacted [47]. Whether similar drug concentration can be found for SA in IELD is what we need to further study. Second, in this study, five patients (4.5%) developed hypotension and three patients (2.7%) complained of respiratory distress. The main reason may be that the change of body position affects the distribution of the local anesthetic and the changes of hemodynamics [48]. The anesthesia level of these patients with adverse reactions was T6-T8. Therefore, choosing a lower puncture point for anesthesia, maintaining supine position longer after anesthesia, preparing vasoactive drugs, oxygen inhalation and proper positioning of chest cushion may be beneficial to prevent high anesthesia level, treat hypotension and respiratory distress. Third, the reported incidence of urinary retention after SA for spinal surgery is 4.3%[49]. The incidence in our study was slightly lower, probably because we asked the patients to empty their bladder before surgery. Finally, lumbar puncture for SA administration using a fine needle is difficult [33] and requires considerable practice. Moreover, SA should not be used in patients whose operation time might be longer than normal. Regardless, all patients should be informed that conversion to GA may be necessary based on intraoperative conditions [8].

This study is limited by its single-center retrospective design and should be considered a preliminary exploration of the safety and efficacy of SA in IELD. Future large-scale randomized controlled trials are warranted to compare IELD under SA, LA, and GA.

## Conclusion

SA can achieve satisfactory pain control for patients undergoing IELD with a low incidence of adverse events. SA may be a useful alternative to LA and GA for IELD surgery. Future randomized controlled trials are warranted to investigate.

## Data Availability

The data supporting the findings of this study are available upon request from the corresponding author (Jinsong Zhao, M.D; Email: 378461861@qq.com).

## Abbreviations

SA: Spinal anesthesia
IELD: Interlaminar endoscopic lumbar discectomy
GA: General anesthesia
LA: Local anaesthesia; POCD: postoperative cognitive dysfunction
ODI: Oswestry disability index
VAS: Visual analog scale
